# Elevated homocysteine as an independent risk for intracranial atherosclerotic stenosis

**DOI:** 10.18632/aging.102019

**Published:** 2019-06-12

**Authors:** Ying Liu, Jing-Hui Song, Xiao-He Hou, Ya-Hui Ma, Xue-Ning Shen, Wei Xu, Fu-Rong Sun, Qiang Dong, Jin-Tai Yu, Lan Tan, Song Chi

**Affiliations:** 1 Department of Neurology, The Affiliated Hospital of Qingdao University, Qingdao, China; 2 Department of Neurology, Qingdao Municipal Hospital, Qingdao University, Qingdao, China; 3 Department of Neurology and Institute of Neurology, Huashan Hospital, Shanghai Medical College, Fudan University, Shanghai, China

**Keywords:** homocysteine, atherosclerosis, intracranial atherosclerotic stenosis, acute ischemic stroke, magnetic resonance angiography

## Abstract

To investigate the association of homocysteine concentration with intracranial atherosclerotic stenosis (ICAS), we assessed 933 acute ischemic stroke patients (346 with ICAS, 587 without ICAS) and 798 non-stroke controls (175 with ICAS, 623 without ICAS) with magnetic resonance angiography (MRA). Homocysteine concentration was found to be significantly higher in participants with ICAS than those without ICAS. In logistic regression analyses, homocysteine concentration was significantly associated with ICAS both in patients (OR: 1.04; 95% CI: 1.01–1.08; P=0.008) and controls (OR: 1.10; 95% CI: 1.06–1.15; P<0.001) for 1 μmol/L increment in homocysteine. Compared with the lowest quartile, the second (OR:1.53; 95% CI: 1.01-2.33), third (OR:1.71; 95% CI: 1.14 -2.60) and fourth (OR:2.48; 95%CI: 1.63-3.81) quartiles were independent predictors of ICAS in patients (P for trend<0.001); the third (OR:1.99; 95% CI: 1.18-3.40) and fourth (OR:2.36; 95%CI: 1.38-4.10) quartiles were independent predictors of ICAS in controls (P for trend<0.001). Hence, elevated homocysteine might be an independent risk for ICAS.

## INTRODUCTION

Stroke has become one of the leading causes of death and disability in China despite the obvious improvement in life expectancy [1]. Intracranial atherosclerotic stenosis (ICAS) is the most common cause of stroke in Asian population and is estimated to account for 33% to 50% of ischemic stroke in China [2]. Identifying the modifiable risk factors of ICAS is essential to the prevention of both occurrence and recurrence of stroke.

Homocysteine (Hcy), a key metabolite of methionine, is proposed to be actively involved in numerous biochemical reactions, with compelling evidences indicating that hyperhomocysteinemia (HHcy) is a candidate risk factor for ischemic stroke [3–6]. Nonetheless, the association between homocysteine and stroke is still controversial [7]. Some studies suggested that Hcy was a risk factor of special subtypes of stroke as it was prone to be higher in stroke with large-artery atherosclerosis [8, 9]. Moreover, HHcy was also found to be associated with poor prognosis, mortality and neurological deterioration after stroke [10–14], especially in the population with large-vessel atherosclerosis [15, 16], which implied the potential role of Hcy in pro-atherosclerotic process. Currently, most studies focus on the association between stroke and Hcy according to Trial of Org 10172 in Acute Stroke Treatment (TOAST) criteria [15–18], with few and inconsistent conclusions on ICAS involving Hcy. Some studies demonstrated that patients with HHcy had severer stenosis and more arterial lesions in the intracranial arteries [19–21]. Conversely, a study of 825 stroke patients showed that the positive association of HHcy and burden of large cerebral arteries existed only in the extracranial arteries [22]. Similarly, another study found that HHcy had no effect on extracranial and intracranial large arteries [23].

Due to the lack of large sample evidence on pro-atherosclerosis of Hcy involving intracranial arteries, we aim to assess the association between HHcy and ICAS in stroke and non-stroke populations as well as to explore the role of Hcy on the number of ICAS segments in China.

## RESULTS

### Characteristics of participants

Totally 1731 participants were included in the study, among whom 933(53.90%) were ischemic stroke patients and 798(46.10%) were controls. There were 346 (19.99%) patients and 175 (10.11%) controls with at least one segment of ICAS, while 587 (33.91%) patients and 623 (35.99%) controls were absent of ICAS. Baseline characteristics were illustrated in Table 1. Compared with controls, patients with acute ischemic stroke were yanger and had higher level of total cholesterol (TC), low density lipoprotein (LDL) and serum glucose (GLU), Hcy, blood pressure and higher proportions of male, history of diabetes mellitus, smoking and drinking but lower proportion of coronary heart disease and antihypertensive treatment. In the patients of ischemic stroke, those with ICAS were older and had higher proportions of male, history of hypertension, diabetes mellitus, coronary heart disease, antihypertensive and antidiabetic treatment as well as higher level of GLU, Hcy, while lower proportions of drinking and level of TC, LDL. In the non-stroke controls, those with ICAS were older and had higher proportions of history of hypertension, diabetes mellitus, and antidiabetic treatment as well as higher level of GLU, Hcy and systolic blood pressure.

**Table 1:** Clinical and demographic characteristics of study cohorts.

	**Acute ischemic stroke patients (N=933)**	**Non-stroke controls (N=798)**	**P**	**Acute ischemic stroke patients**			**Non-stroke controls**		
				**With ICAS(N=346)**	**Without ICAS(N=587)**	**P**	**With ICAS(N=175)**	**Without ICAS(N=623)**	**P**
**Male, no (%)**	610(65.06%)	427(53.51%)	0.027	210(60.69%)	400(68.14%)	0.021	93(53.14%)	334(53.61%)	0.114
**Age(year)**	66.98±11.19	68.18±10.57	0.033	68.53±11.48	66.07±11.39	<0.001	70.57±11.05	67.51±10.57	0.001
**Triglycerides(μmol/L)**	1.30(0.98–1.75)	1.27(0.91–1.77)	0.151	1.26(0.99–1.64)	1.32(0.97–1.86)	0.248	1.38(0.96–1.82)	1.24(0.90–1.76)	0.133
**Total cholesterol(μmol/L)**	5.01(4.31–5.86)	4.75(4.14–5.53)	<0.001	4.89(4.16–5.72)	5.05(4.40–5.92)	0.024	4.74(3.99–5.74)	4.75(4.16–5.48)	0.744
**High density lipoprotein(μmol/L)**	1.11(0.95–1.29)	1.11(0.94–1.33)	0.556	1.10(0.92–1.28)	1.12(0.96–1.29)	0.074	1.07(0.91–1.33)	1.11(0.95–1.35)	0.334
**Low density lipoprotein(μmol/L)**	3.09(2.60–3.69)	2.90(2.40–3.44)	<0.001	3.02 (2.50.–3.56)	3.15(2.67–3.74)	0.026	2.88(2.38–3.50)	2.91(2.43–3.42)	0.734
**Plasma glucose (μmol/L)**	5.47(4.75–7.48)	5.14(4.53–6.32)	<0.001	5.87(4.79–8.11)	5.37(4.72–6.88)	0.003	5.51(4.54–7.57)	5.05(4.53–6.04)	0.001
**Homocysteine(μmol/L)**	9.20(7.00–12.30)	8.50(6.80–11.10)	<0.001	9.75(7.60–13.00)	8.90(7.00–11.88)	0.002	9.70(7.60–12.80)	8.10(6.60–10.60)	<0.001
**Systolic blood pressure**	150.0(135.0–165.0)	140.0(130.0–152.8)	<0.001	150.0(140.0–170.0)	150.0(130.0–164.5)	0.055	150.0(133.0–166.5)	140.0(130.0–150.0)	<0.001
**Diastolic blood pressure**	85.0(80.0–91.0)	80.0(78.0–90.0)	<0.001	85.0(80.0–90.0)	86.0(80.0–95.0)	0.076	82.0(80.0–90.0)	80.0(77.0–90.0)	0.193
**Medical history, n (%)**									
**Hypertension**	723(77.49%)	601(75.31%)	0.287	282(81.50%)	441(75.13%)	<0.001	145(82.86%)	456(73.19%)	0.009
**Diabetes mellitus**	356(38.16%)	263(32.96%)	0.024	157(45.38%)	199(33.90%)	0.019	78(44.57%)	185(29.70%)	<0.001
**Coronary heart disease**	299(32.05%)	354(44.36%)	<0.001	127(36.71%)	172(29.30%)	0.009	82(46.86%)	272(43.66%)	0.452
**Cigarette smoking**	356(38.16%)	229(28.70%)	<0.001	119(34.39%)	237(40.37%)	0.069	59(33.71%)	170(27.29%)	0.097
**Alcohol consumption**	277(29.69%)	153(19.17%)	<0.001	89(25.72%)	188(32.03%)	0.042	42(24.00%)	111(17.82%)	0.066
**Antihypertensive treatment**	278(29.80%)	378(47.37%)	<0.001	132(38.15%)	146(24.87%)	<0.001	81(46.29%)	297(47.67%)	0.745
**Antidiabetic treatment**	158(16.93%)	150(18.80%)	0.313	81(23.41%)	77(13.12%)	<0.001	43(24.57%)	107(17.17%)	0.027

### Association between serum Hcy and ICAS

In unadjusted continuous model, Hcy was significantly associated with ICAS both in stroke patients (OR: 1.03; 95% CI: 1.00-1.06; P=0.044) and controls (OR: 1.10; 95% CI: 1.06-1.14; P<0.001). The adjusted odds ratio of 1 μmol increment in Hcy remained statistically significant in stroke patients (OR: 1.04; 95% CI: 1.01-1.08; P=0.008) and controls (OR: 1.10; 95% CI: 1.06-1.15; P<0.001) (Table 2). We further divided all participants into four groups according to quartiles of Hcy concentration. In unadjusted model, Hcy quartiles 3 and 4 were significantly associated with the presence of ICAS both in stroke patients and controls. After having been adjusted for all potential covariates listed in Table 1, the OR was 1.53 (95% CI 1.01 to 2.33; P=0.045) for the second quartile, 1.71 (95% CI 1.14 to 2.60; P=0.011) for the third quartile, and 2.48 (95% CI 1.63 to 3.81; P<0.001) for the fourth quartile in stroke patients (P for trend<0.001). Correspondingly, in the controls, the OR was 0.93 (95% CI 0.52 to 1.64; P=0.791) for the second quartile, 1.99 (95% CI 1.18 to 3.40; P=0.010) for the third quartile, and 2.36 (95% CI 1.38 to 4.10; P=0.002) for the fourth quartile (P for trend<0.001) (Table 2).

**Table 2:** Multivariate logistic regression analysis of the association between Hcy and ICAS.

		**Crude**		**Mode 1**	
		**OR (95%IC)**	**P**	**OR (95%IC)**	**P**
Acute ischemic stroke patients	Continuous	1.03(1.00–1.06)	0.044	1.04(1.01–1.08)	0.008
	Q1 (≤7.00μmol/L)	ref		ref	
	Q2 (7.01–9.20μmol/L)	1.29(0.87–1.90)	0.201	1.53(1.01–2.33)	0.045
	Q3 (9.21–12.30μmol/L)	1.48(1.01–2.17)	0.047	1.71(1.14–2.60)	0.011
	Q4 (>12.30μmol/L)	1.96(1.34–2.88)	0.001	2.48(1.63–3.81)	<0.001
	P-value for trend		<0.001		<0.001
Non-stroke controls	Continuous	1.10(1.06–1.14)	<0.001	1.10(1.06–1.15)	<0.001
	Q1 (≤6.80μmol/L)	ref		ref	
	Q2 (6.81–8.50μmol/L)	0.91(0.52–1.56)	0.723	0.93(0.52–1.64)	0.791
	Q3 (8.51–11.10μmol/L)	2.08(1.28–3.42)	0.003	1.99(1.18–3.40)	0.010
	Q4 (>11.10μmol/L)	2.38(1.47–3.90)	<0.001	2.36(1.38–4.10)	0.002
	P-value for trend		<0.001		<0.001

Mode 1: Adjustment for gender, age, triglycerides, cholesterol, high-density lipoprotein, low density lipoprotein, plasma glucose, systolic blood pressure, diastolic blood pressure and history of hypertension, diabetes mellitus, coronary heart disease, smoking, drinking, antihypertensive treatment and antidiabetic treatment. OR, odds ratios; 95%CI, 95% confidence interval; ICAS, intracranial stenosis; Hcy, homocysteine.

**Figure 1:**
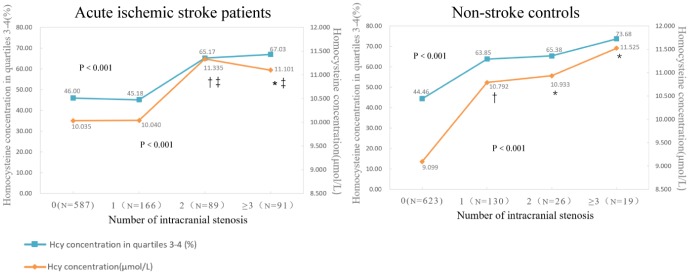
**Compariation of Hcy concentration (μmol/L) and percentages of the third and fourth quarter of Hcy distribution according to the number of ICAS in ischemic stroke patients and non-stroke controls.** Data are mean of Hcy concentration and percentages of patients in third and fourth quartiles. P value indicates the comparison among four groups with Kruskal–Wallis and Chi-squared test respectively. * P < 0.05 in comparison with those absent of ICAS. † P < 0.001 in comparison with those absent of ICAS. ‡ P<0.05 in comparison with those with one segment of ICAS.

In addition, participants with more numbers of stenosed intracranial vessels tended to have higher Hcy concentration (P<0.001) and higher percentage of the third and fourth quarter of Hcy distribution (P<0.001) (Figure 1). As for acute ischemic stroke, patients with more than one segment of ICAS had significantly higher Hcy concentration than those with only one segment or absence of ICAS. In non-stroke controls, significant differences were also observed between those with at least one segment of ICAS and individuals with no ICAS (Table 3). The Hcy concentration between the anterior and posterior circulation was not significantly different (P=0.546).

**Table 3:** P values for tests of differences of Hcy concentration between groups.

In acute ischemic stroke patients	Number of ICAS (Hcy, mean, μmol/L)			
		1(10.040)	2(11.335)	3(11.101)
	0 (10.035)	0.937	0.001	0.002
	1 (10.040)		0.002	0.017
	2 (11.335)			0.656
	3 (11.101)			
In non-stroke controls	Number of ICAS (Hcy, mean, μmol/L)			
		1(10.792)	2(10.933)	3(11.525)
	0 (9.099)	<0.001	0.025	0.007
	1 (10.792)		0.690	0.333
	2 (10.933)			0.573
	3 (11.525)			

## DISCUSSION

The principal finding of this study was that elevated Hcy was independently associated with ICAS irrespective of the stroke condition. Moreover, a dose-response relationship was observed between Hcy concentration and the number of ICAS segments, with higher Hcy correlating with a greater number of ICAS segments.

Previous studies which focused on the association of HHcy and atherosclerosis of large cerebral vessel have obtained conflicting results. Only a few studies conducted in the population of acute cerebral infarction, transient ischemic attack and asymptomatic hypertension, demonstrated that HHcy was associated with advanced intracranial atherosclerosis [19, 24] and the severity of large arteries stenosis [20, 21]. Our findings were largely in line with two previous studies, both of which indicated that HHcy was associated with cerebral arterial stenosis, though the former had limited number of participants, while the latter was conducted in the population of asymptomatic hypertension [21]. Of note, our study strengthened the previous findings that HHcy was associated with the burden of ICAS by establishing a dose-response relationship between HHcy and ICAS. Hence, HHcy plays an essential role in the deterioration of intracranial atherosclerosis. Previous study found that different mechanisms might get involved in the pathogenesis of anterior and posterior circulation ICAS [25]. However, we did not observe significant difference of Hcy between the anterior and posterior ICAS in our data.

Though potential mechanisms of Hcy for ICAS are not fully understood, one major mechanism is that HHcy activates oxidative stress and its downstream signaling pathways, which subsequently leads to vascular inflammation and endothelial dysfunction [26–30]. Moreover, a new survey has found that Hcy can directly activate the angiotensin II type I receptor, which plays a significant part in pathological vascular injuries [31]. Compared with extracranial artery, unaffected intracranial arteries have enhanced antioxidant response. However, this antioxidant protection markedly decreases with increased age. Consequently, atherosclerosis of intracranial arteries is faster than that of extracranial arteries [32]. In addition, in pathological condition, intracranial artery might be more susceptible to inflammatory changes owing to reduced expression of inhibitors of inflammation, and prominent expression of proin-flammatory proteasomes [33].

Previous studies reported that HHcy was more common in stroke due to ICAS than other stroke subtypes [9]. And some case-control studies showed that HHcy was not as strong a risk factor for small-vessel stroke or other stroke subtypes as it was for large-artery atherosclerosis subtype [8, 34]. Similarly, some studies indicated that the correlation between Hcy and mortality after stroke or recurrent ischemic stroke was only significant in the large-artery atherosclerosis [15, 16]. All the studies highlighted that Hcy played an essential role in intracranial vascular injury. Our study further supported the role of HHcy in the large cerebral artery atherosclerosis in the population of acute ischemic stroke and non-stroke controls. Therefore, HHcy might be an important marker of vascular injury and a potential target for vascular injury prevention, especially for the intracranial artery.

### Limitations

There are some limitations in this study. First, we did not evaluate the role of folate and vitamin B6 / B12 which could influence the level of Hcy and bias the association. Second, only MRA was used to estimate the stenosis. It is known that the spatial resolution of MRA is limited; therefore, it is not as accurate as that of catheter angiography in evaluating arterial stenosis. Third, we could not conclude the causal relationship between Hcy and ICAS because the level of Hcy was not assessed before the onset. Fourth, although we have considered many traditional risk factors, there are still some confounding factors that are not included in this study.

### Future directions

More conclusive data from well-designed longitudinal studies and randomized controlled trials are needed to understand the mechanism and long-term effect of Hcy on the ICAS. Moreover, future preclinical studies are needed to determine whether the control of Hcy level is beneficial to vascular injury and the prevention of ischemic stroke, especially those due to ICAS.

## METHODS

### Study design and participants

Participants were prospectively recruited from the Neurology Department and Health & Physical Examination Center of Qingdao Municipal Hospital from January 2014 to June 2018. All are aged over 40 years and have underwent systemic investigations including magnetic resonance imaging (MRI), MRA, and other essential laboratory tests. Acute ischemic stroke is defined as transient ischemic attack (TIA) or MRI confirmed acute ischemic stroke (within 7 days of onset). The non-stroke controls are lack of TIA or acute ischemic stroke that confirmed by neuroimaging. Exclusion criteria included: (1) history of stroke; (2) cardioembolic stroke or evidences of cardioembolic propensity such as history of atrial fibrillation, valvular heart disease and postaortic valve replacement; (3) arterial dissection, arteritis, moyamoya disease and muscle fiber dysplasia; (4) severe stenosis of extracranial carotid artery; (5) serious infection, malignant tumor, chronic liver disease and renal insufficiency. Written informed consents were obtained from all participants or their legal representatives. This study was approved by the Institutional Ethics Committees of Qingdao Municipal Hospital.

### Assessment of intracranial arterial stenosis

Images were obtained by a 3D-TOF MRA (TR/TE=20-25/2-3ms, flip angle 15°, FOV=220mm × 220mm, Matrix 320X224, 1.2 mm section thickness and 0.6 mm slice acquisition interval). The presence of ICAS was defined as 50%-99% stenosis according to the Warfarin-Aspirin Symptomatic Intracranial Disease trial criteria [35] or occlusion in at least one of the following arterial segments: proximal portion of the middle (M1/2), anterior (A1/2), or posterior (P1/2) cerebral artery; the basilar artery; or the intracranial portion of the internal carotid artery or vertebral artery (V4). Two experienced radiologists blind to the clinical information independently performed the assessment. Any disagreement was reviewed by a third reader and adjudicated by consensus.

### Demographic and clinical measurements

Demographic information, vascular risk factors, history of stroke, alcohol consumption, antihypertensive and antidiabetic treatment were collected by trained and certified neurologists. Vascular risk factors mainly included serum lipid and glucose, blood pressure, hypertension, diabetes mellitus, coronary artery disease, and current and history of smoking. Hypertension was defined as an average systolic blood pressure ≥ 140mmHg, an average diastolic blood pressure ≥ 90mmHg or using antihypertensive drugs [36]; Diabetes mellitus was defined as an average level of fasting plasma glucose ≥ 7.0mmol/L, or using anti-diabetic medication [37]; coronary artery disease was defined as a history of coronary artery disease or being newly diagnosed; smoking and drinking were defined as self-reported behaviors of former or current smoking and drinking, respectively.

### Laboratory measurement

Venous blood samples were collected within 12 hours of admission and performed in the Central Laboratory of Qingdao Municipal Hospital using the standard protocols measured with automatic biochemical analyzer BECKMAN COULTER AU5800 (Beckman Coulter Inc. Brea, CA, USA), including serum concentrations of total Hcy, TG, TC, high density lipoprotein (HDL), LDL and GLU.

### Statistical analyses

Continuous variables were described as median with interquartile range or mean with standard deviation (SD), and categorical variables were expressed as number and percentage. We used Wilcoxon test and Kruskal–Wallis test for comparison of non-parametric variables and Chi-squared test for categorical variables. Multivariate logistic regression was used to analyze the association of Hcy with ICAS. The analyses were adjusted for confounding variables that included all the potential covariates listed in Table 1 (Model 1). Results were given by odds ratio (OR) and 95% confidence interval (CI) of each quartile and then median values of each quartile were treated as a continuous variable into model of logistic regression to perform the trend test. A 2-tailed P<0.05 was considered statistically significant. R software, version 3.4.4 (http://R-project.org/), was used for all statistical analyses.

## CONCLUSIONS

In summary, elevated Hcy is significantly associated with ICAS irrespective of the stroke condition, and there is a dose-response relationship between Hcy concentration and the number of ICAS segments. Therefore, HHcy might be an important marker of vascular injury and a potential target for vascular injury prevention, especially for the intracranial artery.

## References

[1] Yang G, Wang Y, Zeng Y, Gao GF, Liang X, Zhou M, Wan X, Yu S, Jiang Y, Naghavi M, Vos T, Wang H, Lopez AD, Murray CJ. Rapid health transition in China, 1990-2010: findings from the Global Burden of Disease Study 2010. Lancet. 2013; 381:1987–2015. 10.1016/S0140-6736(13)61097-1 23746901 PMC7159289

[2] Gorelick PB, Wong KS, Bae HJ, Pandey DK. Large artery intracranial occlusive disease: a large worldwide burden but a relatively neglected frontier. Stroke. 2008; 39:2396–99. 10.1161/STROKEAHA.107.505776 18535283

[3] Casas JP, Bautista LE, Smeeth L, Sharma P, Hingorani AD. Homocysteine and stroke: evidence on a causal link from mendelian randomisation. Lancet. 2005; 365:224–32. 10.1016/S0140-6736(05)70152-5 15652605

[4] Kloppenborg RP, Geerlings MI, Visseren FL, Mali WP, Vermeulen M, van der Graaf Y, Nederkoorn PJ, and SMART Study Group. Homocysteine and progression of generalized small-vessel disease: the SMART-MR Study. Neurology. 2014; 82:777–83. 10.1212/WNL.0000000000000168 24477110

[5] Han L, Wu Q, Wang C, Hao Y, Zhao J, Zhang L, Fan R, Liu Y, Li R, Chen Z, Zhang T, Chen S, Ma J, et al. Homocysteine, Ischemic Stroke, and Coronary Heart Disease in Hypertensive Patients: A Population-Based, Prospective Cohort Study. Stroke. 2015; 46:1777–86. 10.1161/STROKEAHA.115.009111 26038522

[6] Harris S, Rasyid A, Kurniawan M, Mesiano T, Hidayat R. Association of High Blood Homocysteine and Risk of Increased Severity of Ischemic Stroke Events. Int J Angiol. 2019; 28:34–38. 10.1055/s-0038-1667141 30880891 PMC6417904

[7] Verhoef P, Hennekens CH, Malinow MR, Kok FJ, Willett WC, Stampfer MJ. A prospective study of plasma homocyst(e)ine and risk of ischemic stroke. Stroke. 1994; 25:1924–30. 10.1161/01.STR.25.10.1924 8091435

[8] Eikelboom JW, Hankey GJ, Anand SS, Lofthouse E, Staples N, Baker RI. Association between high homocyst(e)ine and ischemic stroke due to large- and small-artery disease but not other etiologic subtypes of ischemic stroke. Stroke. 2000; 31:1069–75. 10.1161/01.STR.31.5.1069 10797167

[9] Gungor L, Polat M, Ozberk MB, Avci B, Abur U. Which Ischemic Stroke Subtype Is Associated with Hyperhomocysteinemia? J Stroke Cerebrovasc Dis. 2018; 27:1921–29. 10.1016/j.jstrokecerebrovasdis.2018.02.033 29661647

[10] Kwon HM, Lee YS, Bae HJ, Kang DW. Homocysteine as a predictor of early neurological deterioration in acute ischemic stroke. Stroke. 2014; 45:871–73. 10.1161/STROKEAHA.113.004099 24448992

[11] Markaki I, Klironomos S, Kostulas K, Sjostrand C. Elevated plasma homocysteine upon ischemic stroke is associated with increased long-term mortality in women. PLoS One. 2017; 12:e0183571. 10.1371/journal.pone.0183571 28846725 PMC5573214

[12] Zhong C, Xu T, Xu T, Peng Y, Wang A, Wang J, Peng H, Li Q, Geng D, Zhang D, Zhang Y, Zhang Y, Gao X, He J, and CATIS Investigation Groups. Plasma Homocysteine and Prognosis of Acute Ischemic Stroke: a Gender-Specific Analysis From CATIS Randomized Clinical Trial. Mol Neurobiol. 2017; 54:2022–30. 10.1007/s12035-016-9799-0 26910818

[13] Yin J, Zhong C, Zhu Z, Bu X, Xu T, Guo L, Wang X, Zhang J, Cui Y, Li D, Zhang J, Ju Z, Chen CS, et al. Elevated circulating homocysteine and high-sensitivity C-reactive protein jointly predicts post-stroke depression among Chinese patients with acute ischemic stroke. Clin Chim Acta. 2018; 479:132–37. 10.1016/j.cca.2018.01.011 29325799

[14] Jindal A, Rajagopal S, Winter L, Miller JW, Jacobsen DW, Brigman J, Allan AM, Paul S, Poddar R. Hyperhomocysteinemia leads to exacerbation of ischemic brain damage: Role of GluN2A NMDA receptors. Neurobiol Dis. 2019; 127:287–302. 10.1016/j.nbd.2019.03.012 30885791 PMC6588434

[15] Shi Z, Guan Y, Huo YR, Liu S, Zhang M, Lu H, Yue W, Wang J, Ji Y. Elevated Total Homocysteine Levels in Acute Ischemic Stroke Are Associated With Long-Term Mortality. Stroke. 2015; 46:2419–25. 10.1161/STROKEAHA.115.009136 26199315 PMC4542568

[16] Shi Z, Liu S, Guan Y, Zhang M, Lu H, Yue W, Zhang B, Li M, Xue J, Ji Y. Changes in total homocysteine levels after acute stroke and recurrence of stroke. Sci Rep. 2018; 8:6993. 10.1038/s41598-018-25398-5 29725064 PMC5934407

[17] Wu GH, Kong FZ, Dong XF, Wu DF, Guo QZ, Shen AR, Cheng QZ, Luo WF. Association between hyperhomocysteinemia and stroke with atherosclerosis and small artery occlusion depends on homocysteine metabolism-related vitamin levels in Chinese patients with normal renal function. Metab Brain Dis. 2017; 32:859–65. 10.1007/s11011-017-9978-3 28261756

[18] Piao X, Wu G, Yang P, Shen J, De A, Wu J, Qu Q. Association between Homocysteine and Cerebral Small Vessel Disease: A Meta-Analysis. J Stroke Cerebrovasc Dis. 2018; 27:2423–30. 10.1016/j.jstrokecerebrovasdis.2018.04.035 29801814

[19] Kim JM, Park KY, Shin DW, Park MS, Kwon OS. Relation of serum homocysteine levels to cerebral artery calcification and atherosclerosis. Atherosclerosis. 2016; 254:200–04. 10.1016/j.atherosclerosis.2016.10.023 27760401

[20] Yoo JH, Chung CS, Kang SS. Relation of plasma homocyst(e)ine to cerebral infarction and cerebral atherosclerosis. Stroke. 1998; 29:2478–83. 10.1161/01.STR.29.12.2478 9836754

[21] Wang Y, Zhang J, Qian Y, Tang X, Ling H, Chen K, Li Y, Gao P, Zhu D. Association of Homocysteine with Aysmptomatic Intracranial and Extracranial Arterial Stenosis in Hypertension Patients. Sci Rep. 2018; 8:595. 10.1038/s41598-017-19125-9 29330520 PMC5766541

[22] Jeon SB, Kang DW, Kim JS, Kwon SU. Homocysteine, small-vessel disease, and atherosclerosis: an MRI study of 825 stroke patients. Neurology. 2014; 83:695–701. 10.1212/WNL.0000000000000720 25031284

[23] Oh SH, Kim NK, Kim HS, Kim WC, Kim OJ. Plasma total homocysteine and the methylenetetrahydrofolate reductase 677C>T polymorphism do not contribute to the distribution of cervico-cerebral atherosclerosis in ischaemic stroke patients. Eur J Neurol. 2011; 18:491–96. 10.1111/j.1468-1331.2010.03188.x 20825473

[24] Lu SS, Xie J, Su CQ, Ge S, Shi HB, Hong XN. Plasma homocysteine levels and intracranial plaque characteristics: association and clinical relevance in ischemic stroke. BMC Neurol. 2018; 18:200. 10.1186/s12883-018-1203-4 30522455 PMC6282283

[25] Kim JS, Nah HW, Park SM, Kim SK, Cho KH, Lee J, Lee YS, Kim J, Ha SW, Kim EG, Kim DE, Kang DW, Kwon SU, et al. Risk factors and stroke mechanisms in atherosclerotic stroke: intracranial compared with extracranial and anterior compared with posterior circulation disease. Stroke. 2012; 43:3313–18. 10.1161/STROKEAHA.112.658500 23160885

[26] Chernyavskiy I, Veeranki S, Sen U, Tyagi SC. Atherogenesis: hyperhomocysteinemia interactions with LDL, macrophage function, paraoxonase 1, and exercise. Ann N Y Acad Sci. 2016; 1363:138–54. 10.1111/nyas.13009 26849408 PMC4801713

[27] Fu Y, Wang X, Kong W. Hyperhomocysteinemia and Vascular Injury: The Advance of Mechanisms and Drug Targets. Br J Pharmacol. 2018; 175:1173–1189. 10.1111/bph.13988 28836260 PMC5867019

[28] Hu Y, Xu Y, Wang G. Homocysteine Levels are Associated with Endothelial Function in Newly Diagnosed Type 2 Diabetes Mellitus Patients. Metab Syndr Relat Disord. 2019. 10.1089/met.2019.0011 31045466

[29] Esse R, Barroso M, Tavares de Almeida I, Castro R. The Contribution of Homocysteine Metabolism Disruption to Endothelial Dysfunction: State-of-the-Art. Int J Mol Sci. 2019; 20:20. 10.3390/ijms20040867 30781581 PMC6412520

[30] Jakubowski H. Homocysteine Modification in Protein Structure/Function and Human Disease. Physiol Rev. 2019; 99:555–604. 10.1152/physrev.00003.2018 30427275

[31] Li T, Yu B, Liu Z, Li J, Ma M, Wang Y, Zhu M, Yin H, Wang X, Fu Y, Yu F, Wang X, Fang X, et al. Homocysteine directly interacts and activates the angiotensin II type I receptor to aggravate vascular injury. Nat Commun. 2018; 9:11. 10.1038/s41467-017-02401-7 29296021 PMC5750214

[32] D’Armiento FP, Bianchi A, de Nigris F, Capuzzi DM, D’Armiento MR, Crimi G, Abete P, Palinski W, Condorelli M, Napoli C, Gronholdt ML. Age-related effects on atherogenesis and scavenger enzymes of intracranial and extracranial arteries in men without classic risk factors for atherosclerosis. Stroke. 2001; 32:2472–79. 10.1161/hs1101.098520 11692003

[33] Qureshi AI, Caplan LR. Intracranial atherosclerosis. Lancet. 2014; 383:984–98. 10.1016/S0140-6736(13)61088-0 24007975

[34] Shimizu H, Kiyohara Y, Kato I, Tanizaki Y, Ueno H, Kimura Y, Iwamoto H, Kubo M, Arima H, Ibayashi S, Fujishima M. Plasma homocyst(e)ine concentrations and the risk of subtypes of cerebral infarction. The Hisayama study. Cerebrovasc Dis. 2002; 13:9–15. 10.1159/000047739 11810004

[35] Samuels OB, Joseph GJ, Lynn MJ, Smith HA, Chimowitz MI. A standardized method for measuring intracranial arterial stenosis. AJNR Am J Neuroradiol. 2000; 21:643–46. 10782772 PMC7976653

[36] Liu LS, and Writing Group of 2010 Chinese Guidelines for the Management of Hypertension. [2010 Chinese guidelines for the management of hypertension]. Zhonghua Xin Xue Guan Bing Za Zhi. 2011; 39:579–615. 10.1038/ajh.2011.248 22088239

[37] American Diabetes Association. (2) Classification and diagnosis of diabetes. Diabetes Care. 2015 (Suppl); 38:S8–16. 10.2337/dc15-S005 25537714

